# The use of technology by seniors with neurocognitive disorders in long-term care: a scoping review

**DOI:** 10.1186/s12877-024-05174-z

**Published:** 2024-07-03

**Authors:** Marie-Soleil Hardy, Chaimaa Fanaki, Camille Savoie

**Affiliations:** Faculty of Nursing Science, Université Laval, Québec, QC G1V 0A6 Canada

**Keywords:** Long-term care, Technology, Neurocognitive disorder, Scoping review, Older adults

## Abstract

**Background:**

To map the current state of knowledge about the use of technology with seniors with neurocognitive disorders in long-term care to foster interactions, wellness, and stimulation.

**Methods:**

Cumulative Index to Nursing and Allied Health Literature (CINAHL Plus); MEDLINE; PsycINFO; Embase and Web of Science were searched in eligible literature, with no limit of time, to describe the current use of technology by seniors with neurocognitive disorders in long-term care. All types of literature were considered except for theses, editorial, social media. This scoping review was built around the recommendations of Peters et al. (2020 version). Three researchers collaborated on the selection of articles and independently reviewed the papers, based on the eligibility criteria and review questions.

**Results:**

The search yielded 3,605 studies, of which 39 were included. Most technology type reported was robotics. Included studies reports different positive effects on the use of such technology such as increase of engagement and positive.

**Conclusion:**

The study highlights different types and potential benefits of technology for long-term care residents with neurocognitive disorders, emphasizing the crucial need for additional research to refine interventions and their use.

## Background

According to the World Health Organization (WHO), the percentage of older adults aged 60 years and over will double by 2050 [1]. Since neurocognitive disorders (NCD) occur in older age, their incidence and prevalence rates are thus on the rise. This trend is more common in low- and middle-income countries and regions [2]. It is estimated that the number of individuals with NCD will double every 20 years and reach over 115 million worldwide, by 2050 [3].

A NCD is a degenerative disease that progresses over time as the individual becomes increasingly dependent in conducting daily and domestic activities. According to the Diagnostic and Statistical Manual of Mental Disorders (DSM-5), a major NCD is mainly characterized by a cognitive decline from a previous level of performance in one or more cognitive areas, eventually interfering with the level of independence during everyday activities [4]. This includes Alzheimer’s disease, frontotemporal lobar degeneration, Lewy body disease and vascular disease.

As the disease progresses, older adults with NCD may need to relocate to long-term care (LTC) homes to obtain the needed assistance and support in their daily activities. About 80% of LTC residents suffer from a NCD [5].

LTC homes are progressively integrating technology, including those oriented for the use of residents suffering from NCD. In their article, Gibson et al. identified three types of available technology for people with NCD. The first type represents all devices that are used *on* people with NCD, to improve provided care, such as telecare, fall detectors, GPS locators, key safes, etc. [6]. Different literature reviews have explored these technologies. For example, a recent scoping review listed 54 studies on technologies linked to NCD care, fall detection, and ambient-assisted living technologies [7]. Another systematic review studied the digital care technologies used in people with NCD living in LTC homes to prevent falls and manage the behavioral and psychological symptoms of NCD [8]. The second type includes devices that are used *by* people with NCD [6]. This type of technology is usually used to provide some support for, and to facilitate their daily activities (e.g., medication dispensers, reminder alarms) [6]. Finally, there are devices that are used *with* people with NCD [6]. This third type fosters technologies that promote interaction between a person with NCD and other people or between the person and the technology itself (e.g., telephones, puzzles and games, electronic games & apps). People with Alzheimer’s disease and other NCD can suffer rapid and deleterious consequences from a lack of stimulation and contact with significant people [9]. Recent studies found that videoconferencing helped seniors stay in contact with their families during the COVID-19 pandemic [9–11]. Research has also examined the use of robotic pets to reduce neuropsychiatric symptoms as well as to improve on well-being and loneliness [12, 13]. Even though the use of technology has greatly increased in LTC since the COVID-19 pandemic, there is a lack of knowledge synthesis (i.e., scoping review, systematic review, or literature review) on technology devices used *with* people with NCD. In fact, LTC home restrictions on visits and activities have led to the deployment of several technologies to stimulate residents with major NCD and preserve contact with family caregivers. Aim of this scoping review is to map the evidence on the use of technology in LTC by the elderly with neurocognitive disorders.

## Research questions

The main question guiding this scoping review is:

What literature is available on the use of technology with seniors with major NCD in LTC to foster interactions, wellness, and stimulation?

## Research sub-questions

1. What type of technology is used by the elderly with neurocognitive disorders in long-term care?
2. What are the key findings on potential effects of the use of technology reported in the included literature?

## Methods

### Design

Considering our research objectives, this scoping review was conducted based on the recommendations of Peters et al. [14]. It is a Nine‐step method comprised of the following stages: 1) Defining and aligning the objectives and questions; 2) Developing and aligning the inclusion criteria with the objectives and questions; 3) Describing the planned approach to search for evidence, including selection, data extraction, and presentation of the evidence; 4) Searching for evidence; 5) Selecting evidence; 6) Extracting evidence; 7) Analysis of evidence; 8) Presentation of results; and 9) Summarizing evidence in relation to the purpose of the review, making conclusions and noting any implications related to the findings [14]. A protocol for the study was developed and a comprehensive search conducted in electronic databases. The protocol was registered on the institutional repository CorpusUL (http://hdl.handle.net/20.500.11794/135363).

### Eligibility criteria

To meet the aim of this scoping review, we selected the literature that meets the Population, Concept and Context (PCC) framework proposed by Peters et al. [14].

#### Population

For the population, we included individuals aged a) 65 years and older, of b) all genders, with c) NCD. The individuals most have a diagnosis of NCD as defined by the DSM-5 [4] and include many types of degenerative disorders, such as Alzheimer’s, frontotemporal lobar degeneration, Lewy body disease, and vascular disease.

#### Concept

In terms of the eligibility criteria for the Concept element, the core concept that was used is technology. The definition proposed by Neal et al. was selected, that is “any device or associated software that is able to communicate over a network or respond to the external environment” (p. 914) [15]. This includes other associated concepts, such as computers, smartphones, phones, electronic tablets, robotic devices, and video games. The technology will need to be used, with or without assistance, by seniors, for recreational purposes, wellness or to foster interactions. All studies that included technologies in the service of care will be excluded.

#### Context

The last element of the framework, i.e., the context criteria, allowed the consideration of studies made in the LTC sector. Literature from all countries will be included.

### Search strategy

The following databases were selected for their relevance to nursing, healthcare, and social care: Cumulative Index to Nursing and Allied Health Literature (CINAHL Plus); MEDLINE; PsycINFO; Embase and Web of Science. A comprehensive search strategy was conducted in the databases by the authors in collaboration with an experienced librarian, using search terms based on the five concepts (see Table 1). Literature in English and French were considered for the database search, because those are the shared and mastered languages of authors. Databases were searched with no limit of time, to describe the current use of technology by seniors of NCD in LTC. All types of literature were considered except for theses, editorial, social media, personal blogs, conference proceedings, books or book chapters and study protocol.

**Table 1:** Database: Medline [OVID] – Search Strategy

1	"technolog*"[Title/Abstract] OR "Computer"[Title/Abstract] OR "Electronic tablets"[Title/Abstract] OR "Cellphone"[Title/Abstract] OR "Phone"[Title/Abstract] OR "IPhone"[Title/Abstract] OR "Smartphone"[Title/Abstract] OR "touch screen"[Title/Abstract] OR "iPad"[Title/Abstract] OR "videoconferenc*"[Title/Abstract] OR "video call*"[Title/Abstract] OR "zoom meeting*"[Title/Abstract] OR "Skype"[Title/Abstract] OR "App"[Title/Abstract] OR "Apps"[Title/Abstract] OR "application*"[Title/Abstract]
2	"Telephone"[MeSH Terms:noexp] OR "Cell Phone"[MeSH Terms] OR "Computers"[MeSH Terms] OR "Videoconferencing"[MeSH Terms] OR "Attitude to Computers"[MeSH Terms]
3	#1 AND #2 [2, 354, 837]
4	"long term care"[Title/Abstract] OR "nursing home*"[Title/Abstract] OR "residential care"[Title/Abstract] OR "Homes for the Aged"[Title/Abstract] OR "long term care"[MeSH Terms] OR "Nursing Homes"[MeSH Terms] OR "Homes for the Aged"[MeSH Terms] OR "Residential Facilities"[MeSH Terms:noexp] OR "Assisted Living Facilities"[MeSH Terms]
5	"alzheimer*"[Title/Abstract] OR "Cognition decline"[Title/Abstract] OR "Cognitive decline"[Title/Abstract] OR "cognitive disorder*"[Title/Abstract] OR "cognition disorder*"[Title/Abstract] OR "Cognitive dysfunction"[Title/Abstract] OR "Cognitive disorder"[Title/Abstract] OR "Cognitive impairment"[Title/Abstract] OR "Dementia"[Title/Abstract] OR "Frontotemporal"[Title/Abstract] OR "Lewy Body"[Title/Abstract] OR "Neurocognitive decline"[Title/Abstract] OR "Neurocognitive dysfunction"[Title/Abstract] OR "Neurocognitive impairment"[Title/Abstract] OR "NCD*"[Title/Abstract] OR "Dementia"[MeSH Terms:noexp] OR "Alzheimer Disease"[MeSH Terms] OR "dementia, vascular"[MeSH Terms] OR "Frontotemporal Dementia"[MeSH Terms:noexp] OR "Lewy Body Disease"[MeSH Terms]
6	#3 AND #4 AND #5 [654]

#### Data management

The literature that emerged from the search strategy was imported into EndNote 20™ software, then transferred to Covidence software. All duplicates were removed using Covidence to proceed with the selection process and to produce a PRISMA flow diagram presenting the search and selection process.

#### Selection process

All titles and abstracts identified in the literature search were screened by the authors (MSH, CS, CF), independently. The full text of articles selected by either author in the initial screening stage were reviewed to select the final list of articles. During the selection process, any disagreements or conflicts between the primary reviewers (CS, CF) were resolved by the principal researcher (MSH). Moreover, references for all the considered articles were hand-searched to identify any relevant reports that may have been missed in the search strategy.

#### Data extraction and synthesis

Data were extracted independently by two authors (CS, CF) and checked by comparing extracted data between both authors to reduce errors and bias [14]. Data extraction templates included information on the first authors and date of the publication; title; country where the studies were conducted; study design; population; type of technology; description of intervention and key findings. Extracted data was reported narratively and summarized in tables. A precise description was made of the link between the data obtained from the eligible articles and our research objective and questions [14]. The process of data extraction was iterative, which means that elements were added as needed as the articles were read. The quality of included studies was not formally assessed, as this is a scoping review. Indeed, Munn et al. note that “an assessment of methodological limitations or risk of bias of the evidence included within a scoping review is generally not performed.” (p. 3) [16].

## Results

**Fig. 1:**
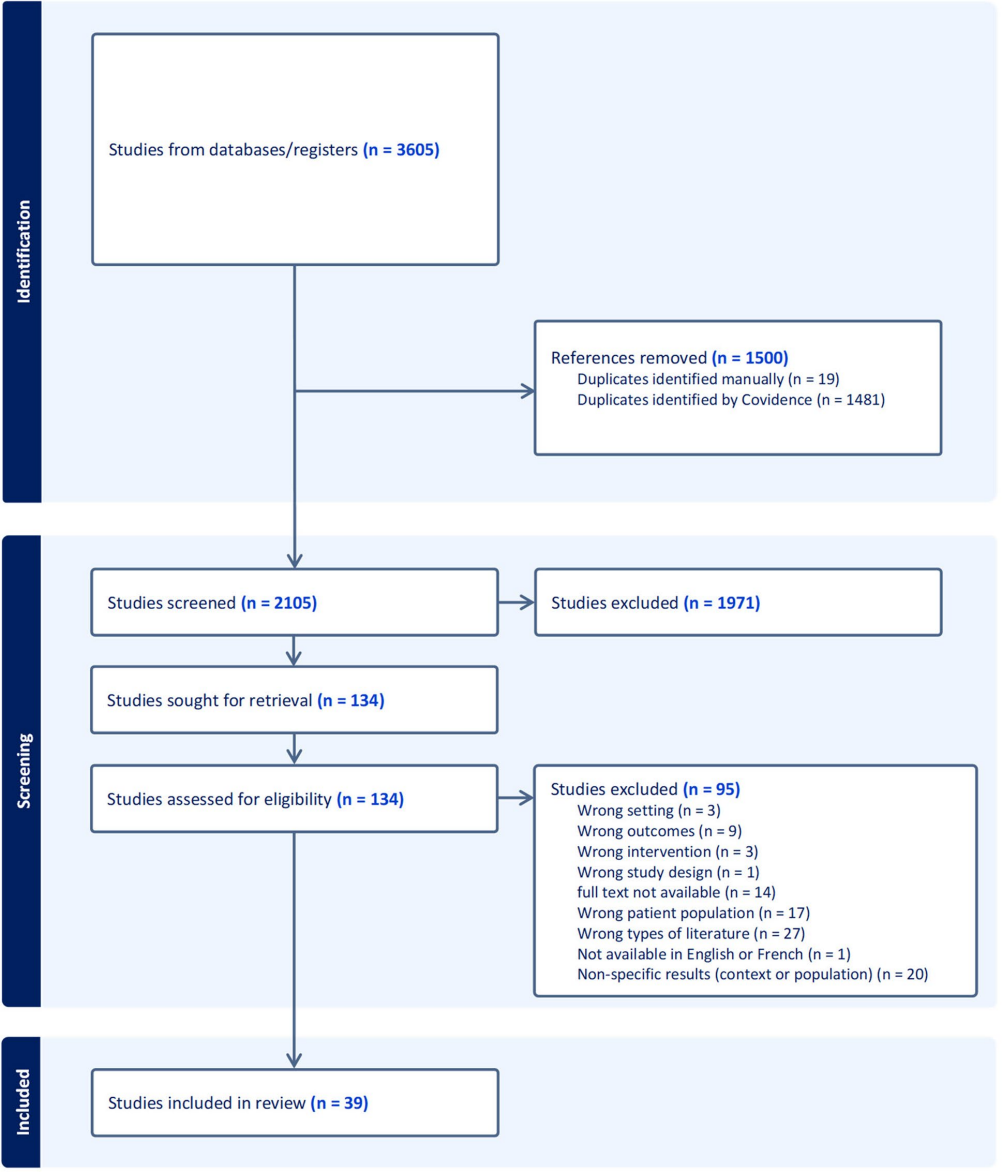
Prisma Flow chart

A total of 3,605 studies were identified after removal of duplicates (Fig. 1). After exclusion of non-relevant results by title and abstract screening, 132 articles were screened by full text and 39 studies were included. Most studies were excluded for not following inclusion criteria on the type of literature, providing nonspecific results or the unavailability of the studies’ full texts.

### Overview of included studies characteristics

The primary studies included in this scoping review took place mainly in Australia and the United States, followed by other countries such as Canada, Ireland, New Zealand, and few European and Asian countries as shown in Table 2.

**Table 2:** Included studies count by country

Authors and country of studies	Study Count
**Australia**	**10**
Chu et al. [17]	
D’Cunha et al. [18]	
Garland et al. [19]	
Khosla et al. [20]	
McAllister et al. [21]	
Moyle et al [22]	
Moyle et al. [23]	
Moyle et al. [24]	
Neal et al. [15]	
Pu et al. [25]	
**Australia, New Zealand, Europe**	**1**
Budak et al. [26]	
**Australia, United States**	**1**
Abraha et al. [27]	
**Canada**	**2**
Hardy et al. [9]	
Sultana et al. [28]	
**China**	**2**
Ke et al. [29]	
Lu et al. [30]	
**Denmark**	**1**
Dinesen et al. [31]	
**Dutch**	**1**
Feng et al. [32]	
**Germany**	**2**
Hoel et al. [33]	
O’Sullivan et al. [34]	
**Ireland**	**2**
Barrett et al. [35]	
Mannion et al. [36]	
**Italy**	**1**
Lancioni et al. [37]	
**Japan**	**2**
Nishiura et al. [38]	
Obayashi et al. [39]	
**Malta**	**1**
Scerri et al. [40]	
**New Zealand**	**1**
Robinson et al. [41]	
**Norway**	**1**
Blindheim et al. [42]	
**Singapore**	**1**
Tulsulkar et al. [43]	
**Taiwan**	**1**
Chiu et al. [44]	
**United States**	**9**
Caserta et al. [45]	
Ford et al. [46]	
Hamel et al. [47]	
Hensel et al. [48]	
Kerssens et al. [49]	
Libin et al. [50]	
Sautter et al. [51]	
Tak et al. [52]	
Tak [53]	
**Total**	**39**

Of the 39 studies included, 24 were quantitative studies, 5 qualitative studies, and 6 mixed methods studies in addition to 2 systematic reviews and 2 scoping reviews. Most participants in the included studies were LTC residents aged over 65 years old with major NCDs and were predominately women. Few studies recruited LTC staff or relatives.

The characteristics of the included studies are presented in Table 3.

**Table 3:** Characteristics of studies included in the review

Authors	Date	Title	Country	Study design and methods	Participants	Intervention and aim	Technology type	Outcomes	Key findings
**Abraha et al.** [27]	2020	Simulated presence therapy for dementia	Australia, United States	Systematic review	Three (3) studies with a total of 144 residents, predominantly women, mean age over 70 years	Audio or videotape recordings that include positive experiences from the resident’s past life and shared memories involving family or close friends	Video/Audiotape	Behavioural and psychological symptoms; Quality of life (QOL)	No conclusions about the efficacy for treating behavioural and psychological symptoms and improving QOL
**Barrett et al.** [35]	2019	Evaluation of a Companion Robot for Individuals with Dementia: Quantitative Findings of the MARIO Project in an Irish Residential Care Setting	Ireland	Quantitative; Quasi- experimental; single group, pre-post intervention	Ten (10) residents, mean age of 83 years. Seven (7) of the subjects are women	A robot MARIO that was used by residents to navigate through and engage in its applications	Robotics	QOL; Depression level; Social Support	Residents can have positive social interactions with MARIO, which alleviate boredom and stimulate sustained engagement. This resulted in increased interactions between residents, care staff, family members, and other residents. No statistical change reported for QOL
**Blindheim et al.** [42]	2022	Promoting activity in long-term care facilities with the social robot Pepper: A pilot study	Norway	Qualitative descriptive	Three (3) residents aged between 79 and 93 years	Pepper, a programmable semi-humanoid robot, explored for its potential contributions to residents’ communal activity and physical exercise	Robotics	Communal activity; Physical activity	Communal activities involving the social robot in terms of physical exercise, joint interaction and social stimulation, and communication between residents, and between residents and employees have increased
**Budak et al.** [26]	2021	Can technology impact loneliness in dementia? A scoping review on the role of assistive technologies in delivering psychosocial interventions in long-term care	Australia, New Zealand, Europe	Scoping review	Twenty (20) studies. Sample sizes ranged from 4 to 415 participants	Social robots, including robotic animals, humanoid robots and telepresence robots and multimedia computer systems	Multiple	QOL; Engagement	Communication and interaction through robotic animals have been shown to have the potential to improve QOL. Multimedia computer systems were found to improve engagement and communication. Nintendo Wii facilitated social engagement and, despite physical and technical limitations, people with dementia were able to use the technology and improve their physical fitness
**Caserta et al.** [45]	2002	Video Respite in an Alzheimer’s care center: Group versus solitary viewing	United States	Quantitative exploratory; Quasi-experimental	Twelve (12) residents, average age of 83.2 years. Ten (10) of the subjects were women	A videotape that reminisces about growing up in the 1920s and 1930s	Video/Audiotape	Verbal and nonverbal responses; Cognitive function	Residents showed an increase in verbal and nonverbal responses during the intervention. The overall level of cognitive impairment did not affect the subjects’ level of participation, attention, interest or enjoyment
**Chiu et al.** [44]	2023	Effects of incorporating virtual reality training intervention into healthcare on cognitive function and well-being in older adults with cognitive impairment: A randomized controlled trial	Taiwan	Quantitative experimental; RTC	Sixty (60) residents, mean age of 80.3 years. 34 were women	Virtual Reality Cognitive Training Intervention utilizing immersive underwater world fish simulation on global cognitive abilities, general cognitive functioning, and QOL in older adults with cognitive impairment in long-term care facilities	Virtual Reality	Cognitive function; QOL	The intervention has significantly improved general cognitive functioning and QOL in the VR group compared to the control group
**Chu et al.** [17]	2017	Service innovation through social robot engagement to improve dementia care quality	Australia	Quantitative; Cross sectional	One hundred and thirty-nine (139) residents aged between 65 and 90 years	Two (2) robots, Sophie and Jack, designed for emotional and intentional communication and interaction purposes with the residents	Robotics	Social engagement	The intervention has significantly improved social engagement and fostered positive interactions
**D’Cunha et al.** [18]	2021	Effects of a virtual group cycling experience on people living with dementia: A mixed method pilot study	Australia	Mixed methods	Ten (10) residents, mean age of 86.1 years. Eight (8) residents were female	Recorded footage for a virtual cycling environment intervention presented to residents on a projector screen	Virtual Reality	Environmental stimulus; EnjoymentApathy; Engagement; Physical activity; Social interactions	Residents responded positively to the intervention. No differences in mood, enjoyment, apathy or engagement
**Dinesen et al.** [31]	2022	Use of a Social Robot (LOVOT) for Persons With Dementia: Exploratory Study	Denmark	Mixed methods	Forty-two (42) residents, mean age of 83,5 years	A social robot LOVOT built with artificial intelligence, to interact with residents in real time and act like a human being	Robotics	Well-being; Mood; Behaviour; Acceptability; Interraction with robot	The intervention had no clinically significant changes in the well-being. Entertaining and calming effects on the residents and promoted communication and social interactions
**Feng et al.** [32]	2022	Context-Enhanced Human–Robot Interaction: Exploring the Role of System Interactivity and Multimodal Stimuli on the Engagement of People with Dementia	Dutch	Quantitative experimental; Crossover	Sixteen (16) residents, mean age of 85.2 years. 12 of the subjects were women	LiveNature is an interactive system design aiming to connect residents to the outdoors through an indoor interactive experience, due to their limited contact with real nature	Virtual Reality	Engagement	The intervention has significantly impacted participants’ engagement, attitude, valence, verbal communications, visual and social engagement
**Ford li et al.** [46]	2019	Evaluating the Impact of Music & Memories Personalized Music and Tablet Engagement Program in Wisconsin Assisted Living Communities: Pilot Study	United States	Quasi-experimental; Pilot study	Thirty-five (35) residents	iPods and iPads used by residents for their app–driven activities such as games, reminiscing about life stories with music, viewing Web-based images or using Google Earth, dabbling in art, and reactivating lifelong learning interests	Tablet	Agitation; QOL; Medication use	The intervention led to a decrease in residents’ agitation and medication use and improved their QOL
**Garland et al.** [19]	2007	A comparison of two treatments of agitated behavior in nursing home residents with dementia: Simulated family presence and preferred music	Australia	Quantitative expérimental; Crossover	Thirty (30) residents, mean age of 79.0 years. 63% were women	Audiotapes of simulated family presence and preferred music	Video/Audiotape	Physical and verbal behaviors	The intervention has significantly decreased residents’ physical and verbal agitated behaviors
**Hamel et al.** [47]	2016	Memory Matters A Mixed-Methods Feasibility Study of a Mobile Aid to Stimulate Reminiscence in Individuals With Memory Loss	United States	Mixed methods	Eighteen (18) residents, mean age of 84 years. Predominately women	Memory Matters was designed to stimulate long-term memories through “cognitive exercise” in which residents physically tap on-screen tiles	Interactive screen	Feasibility and utility	Participants viewed their use favorably and were able to overcome obstacles to use. The intervention has potentially led to enjoyable and beneficial activity for residents, family members, and professional caregivers
**Hardy et al.** [9]	2022	Acceptability of videoconferencing to preserve the contact between cognitively impaired long-term care residents and their family caregivers: A mixed-methods study	Canada	Mixed methods	Thirteen (13) residents, mean age of 82 years. Ten (10) were women	Videoconference meetings through tablets so participants can communicate with their family caregivers	Tablet	Acceptability	The intervention had a good acceptability by residents, increased communication, and positive feelings
**Hensel et al.** [48]	2007	Videophone communication between residents and family: A case study	United States	Qualitative; Case study	Resident 78 years old and her niece 52 years of age	Videophone used by participant to communicate with a relative	Videophone	Experiences of family caregivers and care receivers	The intervention was enjoyable and increased communication between the participant and his/her family member
**Hoel et al.** [33]	2022	Social Health among German Nursing Home Residents with Dementia during the COVID-19 Pandemic, and the Role of Technology to Promote Social Participation	Germany	Mixed methods	Four hundred and seventeen (417) nursing home representatives	Different digital devices to facilitate social participation for residents such as digital music therapy, mobile apps, video games, videoconference, social robots, virtual reality others	Multiple	Social participation; Impacts of Covid-19	Different digital devices used to facilitate social participation for residents with dementia, but there was an apparent preference for digital music therapy
**Ke et al.** [29]	2020	Changes in technology acceptance among older people with dementia: The role of social robot engagement	China	Quantitative, RCT	One hundred and three (103) residents, mean age of 87.2 years	A humanoid robot, Kabochan, to promote communication and engage with residents	Robotics	Technology acceptance; Behavioural engagement	The intervention has potential for changing perceived ease of use but not for other beliefs and attitudes towards technology
**Kerssens et al.** [49]	2014	Managing Dementia Symptoms and Needs Using Technology	United States	Quantitative, Quasi experimental pilot, pre-post trial	Thirty-three (33) residents, mean age of 87 years. Twenty-one (21) of the subjects were women	The Companion is a touch screen computer that delivers psychosocial interventions, such as reminiscence, simulated presence, and orientation to place and time to residents	Interactive screen	Activity of daily life; Sleep; Participation; Mood and behaviors	The intervention has potentially improved difficulties with activities of daily living, sleep, and participation. Health status was perceived as stable
**Khosla et al.** [20]	2017	Human Robot Engagement and Acceptability in Residential Aged Care	Australia	Quantitative, longitudinale	One hundred and fifteen (115) residents aged between 65 and 90 years	Mathilda, a humanoid robot with different applications for entertainment and engaging activities	Robotics	Engagement and acceptability	The intervention has significantly improved residents’ emotional engagement, positive emotion reaction and attitude toward social robots. Improvements in verbal engagement were not statistically significant
**Lancioni et al.** [37]	2013	Self-regulated music stimulation for persons with Alzheimer’s disease: Impact assessment and social validation	Italy	Quantitative, quasi experimental	Ten (10) residents, aged between 78 and 84 years. Eight (8) of the subjects were women	A microswitch, laptop computer, and interface to facilitate active music stimulation, allowing residents to use a simple response and a microswitch to activate music periods during active sessions	Multimedia Computer systems	Participation	The intervention has potentially increased positive participation
**Libin et al.** [50]	2004	Therapeutic robocat for nursing home residents with dementia: Preliminary inquiry	United States	Quantitative, Quasi experimental	Nine (9) relatives of nursing home residents. All were female, mean age of 90 years	A robotic cat, NeCoRo, used to interact with residents	Robotics	Affect; Agitation; Engagement; Cognitive function	The intervention has significantly increased residents’ pleasure, lowered the level of agitation. The level of cognitive functioning was significantly related to the duration of engagement during the intervention
**Lu et al.** [30]	2021	Effectiveness of Companion Robot Care for Dementia: A Systematic Review and Meta-Analysis	China	Systematic Review and Meta-Analysis	Thirteen (13) studies with 20 to 214 residents, predominately women	All different kind of socially assistive robots	Robotics	Agitation; QOL; Depression	The intervention has significantly exhibited changes in agitation. However, no significant changes in depression and QOL were noticed
**Mannion et al.** [36]	2020	Introducing the social robot MARIO to people living with dementia in long-term residential care: Reflections	Ireland	Qualitative descriptive	Seven (7) residents, 4 women and 3 men, aged between 70 and 89 years	A social robot MARIO used by residents to navigate through its applications	Robotics	Level of interest; Engagement	Overall, residents showed positive interest in the intervention, which had a positive effect on their well-being. Residents who had more advanced dementia had greater difficulty using the robot
**McAllister et al.** [21]	2020	Memory Keeper: A prototype digital application to improve engagement with people with dementia in long-term care (innovative practice)	Australia	Qualitative, descriptive pilot study,	Three (3) residents aged 83.76 and 87 years. Two (2) men and 1 woman	The Memory Keeper is a prototype digital application created to present personalised prompts to the person with dementia to stimulate reminiscences	Tablet	Engagement; Feasibility	The intervention was enjoyable and created meaningful engagement between the residents and their family members while supporting their relationship. Increased communication between the participant and his/her family member
**Moyle et al** [22]	2017	Use of a Robotic Seal as a Therapeutic Tool to Improve Dementia Symptoms: A Cluster-Randomized Controlled Trial	Australia	Quantitative, RCT	Four hundred and fifteen (415) residents, predominantly women, mean age of 86 years old	A therapeutic pet robot PARO used to improve residents’ dementia symptoms	Robotics	Engagement; Mood; Agitation	The intervention has significantly impacted participants’ engagement. It helped reduce agitated behaviors. No significant difference detected when it was measured with the Cohen-Mansfield Agitation Inventory Short Form
**Moyle et al.** [24]	2018	Effect of a robotic seal on the motor activity and sleep patterns of older people with dementia, as measured by wearable technology: A cluster-randomised controlled trial	Australia	Mixed methods	One hundred and seventy-five (175) residents for the daytime analyses, and 280 residents for the nighttime analyses. Twenty-eight (28) facilities	A Therapeutic pet-type robotic seal, PARO, has been used as a promising alternative to animal-assisted therapies	Robotics	Daytime and nighttime motor activity; Sleep patterns;	The intervention has helped reduce motor activity. No evidence that it was effective in improving sleep patterns
**Moyle et al.** [23]	2018	Effectiveness of a virtual reality forest on people with dementia: A mixed methods pilot study	Australia	Mixed methods	Ten (10) residents, mean age of 89 years	The “Virtual Reality Forest” is a sensory experience, utilizing a large interactive screen designed to immerse the user in the virtual environment, accompanied by a background soundtrack	Virtual Reality	Mood; Apathy; Engagement	The intervention has significantly increased residents’ pleasure and engagement. However, half of the residents also expressed greater levels of anxiety/fear during the Virtual Reality Forest experience. Not all residents found the Virtual Reality Forest experience to be positive
**Neal et al.** [15]	2020	The use of technology to promote meaningful engagement for adults with dementia in residential aged care: A scoping review	Australia	Scoping review	Twenty (20) studies with 4 to 415 participants. Three (3) studies not reporting the sample size	Different social robots explored for their potential benefit to facilitate residents’ leisure activities	Robotics	Engagement	Engagement not consistent across studies Technologies were reported as providing enjoyable experience, improving well-being and preventing negative outcomes such as agitation or distress
**Nishiura et al.** [38]	2018	Use of a parametric speaker for older people with dementia in a residential care setting: A preliminary study of two (2) cases	Japan	Quantitative, Multiple case study	Two (2) residents. A 78-year-old man and 90-year-old female	A parametric speaker received input from a personal computer to play participant’s favorite pieces of music	Multimedia Computer systems	Behavioral and psychological symptoms of dementia; Cognitive Function	The intervention has significantly decreased residents’ behavioral and psychological symptoms of dementia. Non conclusive results on cognitive function
**Obayashi et al.** [39]	2020	Measuring the impact of age, gender, and dementia on communication-robot interventions in residential care homes	Japan	Non-randomized, multicenter, pre/post-intervention study	Seventy-eight (78) residents, mean age of 86.5 years. 68 of subjects were women	Robots Cota and Palro communicate and interact with people more freely, with a greater degree of freedom and vocabulary	Robotics	QOL; Daily life independence; Functioning; Disability	Improvement in QOL. People with moderate/severe dementia showed greater improvement than those with mild dementia
**O’Sullivan et al.** [34]	2022	A tablet-based intervention for activating nursing home residents with dementia: Results from a cluster-randomized controlled trial	Germany	Quantitative; RCT	One hundred and sixty-two (162) residents with a mean age of 85 years	Tablet-based applications targeting cognitive and functional abilities of residents	Tablet	Apathy; QOL; Medication use	The intervention had no effect on apathy. Improvements in QOL of residents and a reduction of PRN psychotropic medication were observed
**Pu et al.** [25]	2020	The Effect of Using PARO for People Living With Dementia and Chronic Pain: A Pilot Randomized Controlled Trial	Australia	Quantitative Parallel pilot RCT	Forty-three (43) residents, mean of 86 years	A pet robot PARO designed to interact with residents and stimulate them	Robotics	Pain behaviors; Staff-rated pain level; Agitation; Depression; Anxiety	The intervention has shown significant difference in the observational pain change score and significant decrease in PRN medication use. However, no significant difference was observed in staff-rated pain, agitation, anxiety, depression, and regularly scheduled medication
**Robinson et al.** [41]	2013	Suitability of healthcare robots for a dementia unit and suggested improvements	New Zealand	Quantitative Cross sectional	Ten (10) residents aged between 71 and 93 years. Five (5) were women	A pet robot PARO designed to interact with residents and stimulate them	Robotics	Interactions; Engagement; Acceptability	Residents exhibited verbal and non-verbal reactions during the intervention. It was perceived as useful because it is comforting, entertaining, creates interest, and interacts with residents
**Sautter et al.** [51]	2021	Benefits of Computer Engagement in Older Adults with Dementia	United States	Quantitative RCT	Twenty-eight (28) residents	Touch-screen computer platform “It’s Never Too Late” providing the opportunity to personalize computer applications to enhance social connection, facilitate entertainment, and implement cognitive training through various brain fitness programs	Interactive screen	Emotional wellbeing by systolic blood pressure; Behaviors; Medication use	The intervention has significantly improved residents’ well-being and significantly decreased their systolic blood pressure No significant challenging behaviors or the use of antipsychotic medications were observed
**Scerri et al.** [40]	2021	Formal caregivers’ perceptions and experiences of using pet robots for persons living with dementia in long-term care: A meta-ethnography	Malta	Qualitative, meta-ethnography	Eight (8) studies conducted with LTC staff	A pet robot designed to interact with residents and stimulate them	Robotics	Behavioural symptoms; Social connectedness	The intervention was perceived as useful in reducing agitation, restlessness and associated behavioural symptoms, as well as to initiate social connectedness and to evoke old memories and reduce loneliness
**Sultana et al.** [28]	2021	Virtual Reality Experience Intervention May Reduce Responsive Behaviors in Nursing Home Residents with Dementia: A Case Series	Canada	single site case series	Twenty-four (24) residents, mean age of 85.8 years. Most of them were female	Virtual reality experience intervention providing interactive and immersive customized three-dimensional visual and auditory experiences	Virtual Reality	Depression; Medication use	No significant changes in depression or agitation were observed. The dose of prescribed psychotropic drugs was reduced for 8 out of 24 residents after the end of intervention
**Tak et al.** [52]	2015	Computer Activities for Persons with Dementia	United States	Mixed methods	Twenty-seven (27) residents, mean age of 85 years. Predominantly women and Caucasian	Computer activities sessions included email, internet search, computerized games, and slideshow modules	Multimedia Computer systems	Cognitive function	Residents have expressed enthusiasm about the intervention. They exhibited satisfaction and enjoyment through verbal and nonverbal responses
**Tak** [53]	2020	Engaging in Preferred Computer Activities and Cognitive Outcome	United States	Quantiative, descriptive	Twenty-six (26) residents, mean age of 85.2 years. Predominantly women	Different computer activities through a touch screen and different accessories	Multimedia Computer systems	Cognitive function	The increase of time spent during the intervention was significantly correlated with greater improvement in residents’ cognitive function
**Tulsulkar et al.** [43]	2021	Can a humanoid social robot stimulate the interactivity of cognitively impaired elderly? A thorough study based on computer vision methods	Singapore	Quantitative, quasi-experimental,	Fourteen (14) residents	A humanoid robot, Nadine, used to interact with residents inactive/passive for a long time	Robotics	Engagement; Physical activity	Residents have shown differences in the emotions experienced during the intervention with a predominance of neutral expressions

### Technology types and their aim of use

We categorised the technology used in different interventions into 10 major types, as shown in Table 4.

**Table 4:** Count of studies per technology type

Technology type	Count of Study
Robotics	17
Virtual Reality	5
Tablet	4
Multimedia Computer systems	4
Video/Audiotape	3
Interactive screen	3
Multiple	2
Videophone	1
Total	**39**

Most of the included studies utilized robotics [15, 17, 20, 22, 24, 25, 29, 31, 35, 36, 40–43, 50]. This category encompasses 2 types of social robots that were employed in these studies: humanoid or pet robot. These robots were used mainly to stimulate the cognitive and physical abilities of residents through integrated applications. They were also used to provide therapeutic emotional and intentional communication through motion recognition, vocalization, gestures, emotive expressions, singing, or dancing.

Virtual reality was the second most popular technology. Five studies [18, 23, 28, 32, 44] employed software and hardware to create interactive immersive environments presented through video games or 3D videos/images that can be accompanied by music. This technology was used to stimulate residents and improve their cognitive functions, such as psychomotor abilities and memory; to promote physical activity; to reduce responsive behaviors; to increase pleasure or visual and social engagement, especially in a COVID-19 context, as showcased in one of the studies.

Tablets such as iPads were also commonly used. Four studies [9, 34, 40, 46] employed tablet software app–driven activities such as games, music, images, etc. to stimulate reminiscences; to increase pleasure and engagement and to permit residents to communicate with their families and loved one’s through videoconferences.

Four studies [33, 34, 43, 52] used multimedia computer systems that consisted of computer-based interventions with the integration of other software and hardware parts and accessories. Computer activities included email, internet search, games, and slideshow modules or simply broadcasting music played on a laptop through a speaker. They aimed to promote cognitive stimulation and positive emotions for residents, or simply exposing residents to music.

Video/Audiotapes, and interactive screens were reported in three studies, each. Video/audiotapes in these studies [19, 27, 45] consisted of personalized audio or videotape recordings of either simulated family presence, preferred music to treat behavioural and psychological symptoms and improve quality of life of residents with NCD or to stimulate their reminiscence and memory. Interactive screens consisted of the use of computers or projecting screens without the use of keyboard or mouse by the resident to take part in different in-app activities, such as games or audiovisual programs and shows, to stimulate reminiscence and memory and improve or enhance social connection, facilitate entertainment, and implement cognitive training through various brain fitness programs. A scoping review focused on assistive technologies, primarily social robots, and computer systems, to deliver psychosocial interventions; and another study explored social participation among residents with NCD through various digital devices, including mobile apps, video games, video conferencing, social robots, VR technology, and more.

One study utilized a videophone, a telephone device transmitting and receiving a visual image, as well as the sound through the handset, to permit residents to communicate with their family and loved ones, on a weekly basis.

### Overview of study findings on potential effects of technology use to foster interactions, wellness, and stimulation

The review identified a diverse range of technology-based interventions implemented in long-term care facilities for residents with NCD. Table 3 showcased interventions with different technologies explored various outcomes including behavioural and psychological symptoms, cognitive function, quality of life, social and behavioral engagement, medication use, physical and motor activity, daily life activities, sleep patterns etc.

In general, engagement, be it emotional or social or behavioral, was the most frequently reported outcome across various technology interventions. Technologies such as robotics and virtual reality were commonly associated with improvements in engagement levels among seniors with NCD in LTC settings [15, 17, 20, 22, 23, 29, 32, 35, 36, 41, 43, 50]. Another reported no significant impact on this outcome [18]. Quality of life was also a prominent measured outcome [27, 30, 34, 35, 39, 44, 46]. Technologies such as robotics, virtual reality, and tablets were frequently associated with improvements in residents' overall quality of life [25, 26, 39, 40, 42, 44, 46]. While others reported inconclusive results regarding it [27, 30, 35]. Few other studies examined also cognitive function outcome [18, 38, 44, 45, 52, 53]. Interventions using multimedia computer systems and virtual reality were particularly noted for their potential to improve cognitive functioning among seniors with NCD in LTC [44, 52, 53].

Several other key findings emerged, shedding light on various aspects of technology's impact on older adults' well-being and foster positive interactions. For instance, interventions were observed to have varying effects on agitation levels among residents, with some showing reductions [19, 30, 40, 46, 50], meanwhile a study reported no significant changes [22]. Similarly, few interventions were found to impact overall behavioral and psychological symptoms of dementia (BPSD) demonstrating a decrease in these symptoms [19, 22, 38, 40, 46], while others showed inconclusive results on this outcome [27]. Other studies have shown the potential for some technology to enhance communication among residents and their caregivers [21, 26, 31, 32, 41, 42, 48]. Furthermore, physical activity outcomes were examined highlighting the potential for technology interventions to promote physical activity and exercise among seniors with NCD [18, 42, 43]. These findings, in conjunction with those related to engagement, cognitive function, and quality of life, underscore the multifaceted benefits of technology interventions in enhancing various aspects of residents with NCDs well-being and functioning in LTC environments.

## Discussion

This comprehensive scoping review delves into the landscape of technological interventions in LTC for older adults with NCD. Among 3,605 screened articles, 39 met the selection criteria, offering a diverse array of insights. These studies’ geographical distribution indicates a predominant focus on the North American and Australian continents, notably in the United States and Australia, compared to fewer studies originating from Europe, Asia and other countries like Canada, etc. The emergence of Australia and the United States as key locations for research raises intriguing questions that require further exploration to ascertain the underlying factors driving this concentration. Potential factors such as the origins of authors, research infrastructure, funding availability, and regional expertise may contribute to this trend, but a conclusive determination remains elusive, without more detailed investigation. However, a significant disparity becomes apparent in the underrepresentation of low-income countries, even considering the extensively documented increase in the prevalence of NCD in low-income and middle-income countries [54]. This presents a notable ethical concern within research, as well as an opportunity to advance future research endeavors for the development and use of universally applicable technological interventions.

A detailed examination of the technologies used reveals a diverse array of devices used *with* people to foster interactions, wellness, and stimulations. Robotics, encompassing social humanoid and pet robots, emerged as the most frequently studied technology [15, 17, 20, 22, 24, 25, 29, 31, 35, 36, 40–43, 50]. The pre-eminence of robotics in the studied interventions indicates a growing interest in leveraging advanced technologies. These social robots were mainly used to provide cognitive and physical stimulation, emotional communication, and therapeutic engagement. A recent scoping review has presented growing evidence that supports the potential of these technologies to improve the well-being of elderly individuals living in assisted care [55]. Virtual reality’s prominence as the second most reported technology indicates the revolutionary potential of immersive environments for residents with NCD living in LTC to enable them to experience the world. The varied applications, from enhancing cognitive functions to promoting physical activity, showcase this technology’s versatility [18, 23, 28, 32, 44]. A study highlighted the potential of virtual reality interventions in mitigating social isolation among LTC residents [56]. The immersive and interactive nature of virtual reality experiences, as suggested by Hung et al. [56], could offer a novel approach to enhance social engagement and alleviate feelings of loneliness. Additionally, the study emphasizes the importance of considering the specific needs and cognitive abilities of residents when implementing such technologies, especially since a lot of people living with NCD have shown great interest in using this technology [56]. Tablets, with their user-friendly interfaces and portability, emerged as valuable tools to stimulate reminiscence and facilitate communication with families. The adoption of tablets in LTC settings aligns with the broader trend of integrating consumer technologies into healthcare.

The main findings of the objective and effects of the use of technology with seniors with NCD in different studies align with the broader goals of LTC, emphasizing positive interactions, reduced isolation, and increased engagement. While most studies report positive effects, the limited quantity and quality of available research requires caution in drawing overarching conclusions. The diverse study designs and outcomes challenge direct comparisons, emphasizing the need for standardized methodologies and outcome measures for future research. For instance, the focus on residents’ engagement and pleasure aligns with the person-centered care approach, recognizing the importance of individual experiences and preferences [57]. The call for more comprehensive research on the impact of various technologies in reducing isolation and loneliness among residents in LTC facilities echoes the growing recognition of technology as a potential solution to address the social and emotional well-being of older adults [58]. A systematic review has acknowledged the positive effects of technology in improving the overall quality of life for older individuals [59]. However, a literature gap persists concerning the optimal selection of technology, its recommended duration of use, and the appropriate mode of utilization, considering the characteristics of the residents and stages of NCD. To bridge this gap, forthcoming research should prioritize investigating the intricate relationships between technology use and the effects on residents. It is crucial to consider the distinct challenges associated with various stages of NCD, particularly found in individuals with major NCD. This clientele, which is prevalent in LTC, is often excluded from research studies, due to ethical concerns [60]. This creates a knowledge void in understanding the specific needs of these residents and the care required to meet them. The ethical considerations surrounding research involving LTC residents with NCD cannot be overstated. This population’s vulnerability requires a thoughtful and ethical approach to ensure their well-being [60].

### Limits of the study

In acknowledging the limitations of this scoping review, it is essential to highlight that no formal process was employed to assess the quality of the included studies. While every effort was made to select relevant and reliable literature, the absence of a quality assessment process may introduce some degree of uncertainty regarding the robustness of the findings. Furthermore, the significant heterogeneity observed in both the outcomes and methodologies across the included studies presents a challenge. This diversity limits the comparability of findings and underscores the complexity of synthesizing results. As such, caution must be exercised in making overarching conclusions about the true effectiveness of the reported technologies. These limitations underscore the need for future research to employ more rigorous methodologies and standardize outcome measures to facilitate more reliable assessments of technological interventions.

### Implications for future research

This scoping review identifies several gaps and areas for future research in the realm of technological interventions with LTC residents with NCD. The limited geographical diversity of studies calls for broader global representation to account for cultural and contextual variations.

The predominance of robotics in the current literature highlights the need for research exploring the optimal integration of different technologies. Studies assessing the feasibility and effectiveness of various technological modalities with residents at different stages of NCD could further guide LTC managers and practitioners in the selection of technologies and orient their use to achieve desired outcomes.

Further investigations into the effects of these technologies among LTC residents are imperative through standardized methodologies and outcome measures to provide direct comparison.

## Conclusion

The use of technology with residents in LTC facilities shows promise in enhancing socialization, reducing loneliness, and improving quality of life. However, further research may be needed to fine-tune and adjust the interventions. In-depth studies addressing the specific needs of individuals at different stages of NCD, coupled with robust ethical considerations, would not only contribute to academic discussions, but also offer valuable guidance in the LTC field for the well-being of seniors.

## Data Availability

Data supporting the findings of this study are available in the article.

## Abbreviations

DSM: Diagnostic and Statistical Manual of Mental Disorders
LTC: Long-term Care
NCD: Neurocognitive Disorders
RCT: Randomized Controlled Trial
QOL: Quality of life
VR: Virtual Reality

## References

[1] World Health Organization. Ageing and health. 2022. Available from: https://www.who.int/news-room/fact-sheets/detail/ageing-and-health. Cited 2024 Jan 29.

[2] Gao Y, Liu X (2021). Secular trends in the incidence of and mortality due to Alzheimer’s disease and other forms of dementia in China from 1990 to 2019: an age-period-cohort study and joinpoint analysis. Front Aging Neurosci..

[3] McDonald WM (2017). Overview of neurocognitive disorders. Focus (Am Psychiatr Publ).

[4] American Psychiatric Association (2022). Diagnostic and Statistical Manual of Mental Disorders. Diagnostic and Statistical Manual of Mental Disorders.

[5] Association québécoise d'établissements de santé et de services sociaux. Pour la qualité de vie des personnes hébergées en CHSLD : mémoire présenté à la Commission de la santé et des services sociaux dans le cadre de la consultation sur les conditions de vie des adultes hébergés en centre d’hébergement et de soins de longue durée. BAnQ numérique. 2014. Available from: https://numerique.banq.qc.ca/patrimoine/details/52327/2456617. Cited 2024 Jan 29.

[6] Gibson G, Newton L, Pritchard G, Finch T, Brittain K, Robinson L (2014). The provision of assistive technology products and services for people with dementia in the United Kingdom. Dementia (London).

[7] Gettel CJ, Chen K, Goldberg EM (2021). Dementia care, fall detection, and ambient-assisted living technologies help older adults age in place: a scoping review. J Aging Health.

[8] Chan DKY, Chan LKM, Kuang YM, Le MNV, Celler B (2022). Digital care technologies in people with dementia living in long-term care facilities to prevent falls and manage behavioural and psychological symptoms of dementia: a systematic review. Eur J Ageing.

[9] Hardy MS, Fanaki C, Savoie C, Dallaire C, Wilchesky M, Gallani MC (2022). Acceptability of videoconferencing to preserve the contact between cognitively impaired long-term care residents and their family caregivers: A mixed-methods study. Geriatr Nurs (Minneap).

[10] Noone C, McSharry J, Smalle M, Burns A, Dwan K, Devane D (2020). Video calls for reducing social isolation and loneliness in older people: A rapid review. Cochrane Database Syst Rev.

[11] Tsai HH, Cheng CY, Shieh WY, Chang YC (2020). Effects of a smartphone-based videoconferencing program for older nursing home residents on depression, loneliness, and quality of life: A quasi-experimental study. BMC Geriatr.

[12] Petersen S, Houston S, Qin H, Tague C, Studley J (2017). The utilization of robotic pets in dementia care. J Alzheimers Dis.

[13] Van Orden KA, Bower E, Beckler T, Rowe J, Gillespie S (2022). The use of robotic pets with older adults during the COVID-19 pandemic. Clin Gerontol.

[14] Peters MDJ, Marnie C, Tricco AC, Pollock D, Munn Z, Alexander L (2020). Updated methodological guidance for the conduct of scoping reviews. JBI Evid Synth.

[15] Neal I, Du Toit SHJ, Lovarini M (2020). The use of technology to promote meaningful engagement for adults with dementia in residential aged care: A scoping review. Int Psychogeriatr.

[16] Munn Z, Peters MDJ, Stern C (2018). Systematic review or scoping review? Guidance for authors when choosing between a systematic or scoping review approach. BMC Med Res Methodol.

[17] Chu MT, Khosla R, Khaksar SM, Nguyen K (2017). Service innovation through social robot engagement to improve dementia care quality. Assist Technol.

[18] D’Cunha NM, Isbel ST, Frost J, Fearon A, McKune AJ, Naumovski N (2021). Effects of a virtual group cycling experience on people living with dementia: A mixed method pilot study. Dementia.

[19] Garland K, Beer E, Eppingstall B, O’Connor DW (2007). A comparison of two treatments of agitated behavior in nursing home residents with dementia: Simulated family presence and preferred music. American J Geriatr Psychiatry.

[20] Khosla R, Nguyen K, Chu MT (2017). Human robot engagement and acceptability in residential aged care. Int J Hum Comput Interact.

[21] McAllister M, Dayton J, Oprescu F, Katsikitis M, Jones CM (2020). Memory keeper: A prototype digital application to improve engagement with people with dementia in long-term care (innovative practice). Dementia.

[22] Moyle W, Jones CJ, Murfield JE, Thalib L, Beattie ERA, Shum DKH (2017). Use of a robotic seal as a therapeutic tool to improve dementia symptoms: a cluster-randomized controlled trial. J Am Med Dir Assoc.

[23] Moyle W, Jones C, Dwan T, Petrovich T (2018). Effectiveness of a virtual reality forest on people with dementia: a mixed methods pilot study. Gerontologist.

[24] Moyle W, Jones C, Murfield J, Thalib L, Beattie E, Shum D (2018). Effect of a robotic seal on the motor activity and sleep patterns of older people with dementia, as measured by wearable technology: A cluster-randomised controlled trial. Maturitas.

[25] Pu L, Moyle W, Jones C, Todorovic M (2020). The effect of using PARO for people living with dementia and chronic pain: a pilot randomized controlled trial. J Am Med Dir Assoc.

[26] Budak KB, Atefi G, Hoel V (2023). Can technology impact loneliness in dementia? A scoping review on the role of assistive technologies in delivering psychosocial interventions in long-term care. Disabil Rehabil Assist Technol.

[27] Abraha I, Rimland JM, Lozano-Montoya I, Dell’Aquila G, Vélez-Díaz-Pallarés M, Trotta FM (2020). Simulated presence therapy for dementia. Cochrane Database Syst Rev.

[28] Sultana M, Campbell K, Jennings M, Montero-Odasso M, Orange JB, Knowlton J (2021). Virtual reality experience intervention may reduce responsive behaviors in nursing home residents with dementia: a case series. J Alzheimers Dis.

[29] Ke C, Lou VW, Tan KC, Wai MY, Chan LL (2020). Changes in technology acceptance among older people with dementia: the role of social robot engagement. Int J Med Inform..

[30] Lu LC, Lan SH, Hsieh YP, Lin LY, Lan SJ, Chen JC (2021). Effectiveness of companion robot care for dementia: a systematic review and meta-analysis. Innov Aging.

[31] Dinesen B, Hansen HK, Grønborg GB, Dyrvig AK, Leisted SD, Stenstrup H, Skov Schacksen C, Oestergaard C (2022). Use of a social robot (LOVOT) for persons with dementia: exploratory study. JMIR Rehabil Assist Technol..

[32] Feng Y, Perugia G, Yu S, Barakova EI, Hu J, Rauterberg GWM (2022). Context-enhanced human-robot interaction: exploring the role of system interactivity and multimodal stimuli on the engagement of people with dementia. Int J Soc Robot.

[33] Hoel V, Seibert K, Domhoff D, Preuß B, Heinze F, Rothgang H, Wolf-Ostermann K (2022). Social health among german nursing home residents with dementia during the COVID-19 pandemic, and the role of technology to promote social participation. Int J Environ Res Public Health..

[34] O'Sullivan JL, Lech S, Gellert P, Grittner U, Voigt-Antons JN, Möller S, Kuhlmey A, Nordheim J (2022). A tablet-based intervention for activating nursing home residents with dementia: results from a cluster-randomized controlled trial. Int Psychogeriatr.

[35] Barrett E, Burke M, Whelan S (2019). Evaluation of a companion robot for individuals with dementia: quantitative findings of the MARIO project in an irish residential care setting. J Gerontol Nurs.

[36] Mannion A, Summerville S, Barrett E, Burke M, Santorelli A, Kruschke C (2020). Introducing the social robot MARIO to people living with dementia in long term residential care: reflections. Int J Soc Robot.

[37] Lancioni GE, Singh NN, O'Reilly MF, Green VA, Ferlisi G, Ferrarese G, Zullo V, Perilli V, Cassano G, Cordiano N, Pinto K, Zonno N (2013). Self-regulated music stimulation for persons with Alzheimer's disease: impact assessment and social validation. Dev Neurorehabil.

[38] Nishiura Y, Hoshiyama M, Konagaya Y (2018). Use of parametric speaker for older people with dementia in a residential care setting: A preliminary study of two cases. Hong Kong J Occup Ther.

[39] Obayashi K, Kodate N, Masuyama S (2020). Measuring the impact of age, gender and dementia on communication-robot interventions in residential care homes. Geriatr Gerontol Int.

[40] Scerri A, Sammut R, Scerri C (2021). Formal caregivers' perceptions and experiences of using pet robots for persons living with dementia in long-term care: A meta-ethnography. J Adv Nurs.

[41] Robinson H, MacDonald BA, Kerse N, Broadbent E (2013). Suitability of healthcare robots for a dementia unit and suggested improvements. J Am Med Dir Assoc.

[42] Blindheim K, Solberg M, Hameed IA, Alnes RE (2023). Promoting activity in long-term care facilities with the social robot Pepper: a pilot study. Inform Health Soc Care.

[43] Tulsulkar G, Mishra N, Thalmann NM, Lim HE, Lee MP, Cheng SK (2021). Can a humanoid social robot stimulate the interactivity of cognitively impaired elderly? A thorough study based on computer vision methods. Visual Computer.

[44] Chiu HM, Hsu MC, Ouyang WC (2023). Effects of incorporating virtual reality training intervention into health care on cognitive function and well-being in older adults with cognitive impairment: A randomized controlled trial. Int J Hum Comput Stud..

[45] Caserta MS, Lund DA (2003). Video respite® in an Alzheimer's care center. Act Adapt Aging.

[46] Ford JH, Dodds D, Hyland J, Potteiger M (2019). Evaluating the impact of music and memory’s personalized music and tablet engagement program in Wisconsin assisted living communities: pilot study. JMIR Aging.

[47] Hamel AV, Sims TL, Klassen D, Havey T, Gaugler JE (2016). Memory matters: a mixed-methods feasibility study of a mobile aid to stimulate reminiscence in individuals with memory loss. J Gerontol Nurs.

[48] Hensel BK, Parker-Oliver D, Demiris G (2007). Videophone communication between residents and family: a case study. J Am Med Dir Assoc.

[49] Kerssens C, Sattler M, Monteiro A (2014). Managing dementia symptoms and needs using technology. J Gerontol Nurs.

[50] Libin A, Cohen-Mansfield J (2004). Therapeutic robocat for nursing home residents with dementia: preliminary inquiry. Am J Alzheimers Dis Other Demen.

[51] Sautter SW, Ord AS, Azher A, Chidester A, Aravich PF (2021). Benefits of computer engagement in older adults with dementia. Gerontol Geriatr Med..

[52] Tak SH, Zhang H, Patel H, Hong SH (2015). Computer activities for persons with dementia. Gerontologist.

[53] Tak SH (2020). Engaging in preferred computer activities and cognitive outcome. Am J Recreat Ther.

[54] Livingston G, Huntley J, Sommerlad A, Ames D, Ballard C, Banerjee S, Brayne C, Burns A, Cohen-Mansfield J, Cooper C, Costafreda SG, Dias A, Fox N, Gitlin LN, Howard R, Kales HC, Kivimäki M, Larson EB, Ogunniyi A, Orgeta V, Ritchie K, Rockwood K, Sampson EL, Samus Q, Schneider LS, Selbæk G, Teri L, Mukadam N (2020). Dementia prevention, intervention, and care: 2020 report of the lancet commission. Lancet.

[55] Trainum K, Tunis R, Xie B, Hauser E (2023). Robots in assisted living facilities: scoping review. JMIR Aging..

[56] Hung L, Mann J, Wallsworth C, Upreti M, Kan W, Temirova A, Wong KLY, Ren H, To-Miles F, Wong J, Lee C, Kar Lai So D, Hardern S (2023). Facilitators and barriers to using virtual reality and its impact on social engagement in aged care settings: a scoping review. Gerontol Geriatr Med..

[57] McCormack B (2003). A conceptual framework for person-centred practice with older people. Int J Nurs Pract.

[58] Balki E, Hayes N, Holland C (2022). Effectiveness of technology interventions in addressing social isolation, connectedness, and loneliness in older adults: systematic umbrella review. JMIR Aging..

[59] Vailati Riboni F, Comazzi B, Bercovitz K (2020). Technologically enhanced psychological interventions for older adults: a scoping review. BMC Geriatr.

[60] Chandra M, Harbishettar V, Sawhney H, Amanullah S (2021). Ethical issues in dementia research. Indian J Psychol Med.

